# An Infrared Defect Sizing Method Based on Enhanced Phase Images

**DOI:** 10.3390/s20133626

**Published:** 2020-06-28

**Authors:** Yanjie Wei, Zhilong Su, Shuangshuang Mao, Dongsheng Zhang

**Affiliations:** 1 Shanghai Institute of Applied Mathematics and Mechanics, School of Mechanics and Engineering Science, Shanghai University, Shanghai 200444, China; 2278653600@shu.edu.cn (Y.W.); zhilong8845@shu.edu.cn (Z.S.); shshmao@shu.edu.cn (S.M.); 2 Shanghai Key Laboratory of Mechanics in Energy Engineering, Shanghai University, Shanghai 200072, China

**Keywords:** infrared thermography, image enhancement, composite structures

## Abstract

Infrared thermography (IRT) is a full-field, contactless technique that has been widely used for nondestructive evaluation of structural materials due to many advantages. One of the major limitations of IRT is the fuzzy edge and low contrast in the inspected images—as well as the cost of the system. An efficient image post-processing with an affordable and portable device is of great interest to the engineering society. In this study, a convenient and economical inspection system using common halogen lamps was constructed. The corresponding image-processing scheme, which includes Fourier phase analysis and specific image enhancement was developed to identify defects with sharp and clear edges and good contrast. This system was applied to localized of defects in glass-fiber-reinforced composite panels. The results showed that defects with an effective diameter as small as 5 mm can be detected with excellent image quality. As a conclusion, the developed system provides an economic alternative to traditional infrared thermography which is able to identify defects with good qualities.

## 1. Introduction

Infrared thermography (IRT)—a useful non-destructive evaluation (NDE) method—detects subsurface anomalies by analyzing temperature variation at the surface of structures. Active infrared thermography is commonly used in engineering [1,2]. These methods are classified as pulse infrared thermography (PT), lock-in thermography (LT) and long pulse thermography (LPT) [3,4,5] by means of different heating schemes. LPT utilizes low power heating devices such as halogen lamps to excite the object surface for a short period of time to ensure a certain amount of heat was delivered to the object [6,7]. As the external heat transmits into the inside of the object, it produces a thermal contrast gradient during the transient response in areas with subsurface anomalies, which makes it possible to detect subsurface defects. Since the thermal contrast between abnormal and normal regions changes over time, a set of image sequence is recorded and the detected image when the contrast reaches the maximum can be used to identify defects and estimate the corresponding depth underneath the surface [8,9]. Almond et al. [5] applied LPT to metals and composite materials and compared the raw images with PT. It was found that the signal/noise ratio of the raw images acquired with LPT were better than PT. They concluded that LPT was capable to detect defects at deep regions with low noise. It is worth note that heat transfer on the object surface may possess multiple modes, i.e., heat conduction either along the depth or lateral directions in the material and heat convection or radiation with the ambient air. The principle of PT is based on the former by only considering heat conduction within the materials. The raw image acquired for defects located at deep regions are usually blurred due to the heat diffusion [10,11]. In this situation, image processing is expected to obtain high contrast images. For example, Wang et al. [12] applied some signal processing algorithms to the sequential infrared images in order to improve the quality of the image by suppressing noises. They reported that absolute thermal contrast (ATC), thermographic signal reconstruction (TSR), pulse phase thermography (PPT) and principal component analysis (PCA) are helpful for detecting small defects even in deep regions [13,14,15,16]. With the development of infrared detectors, the sensitivity of temperature is much improved to provide a wide dynamic range and high precision. For displaying purpose, an eight-bit image is usually expected in inspection, which may lose information if a simple linear stretching by the maximum and minimum values.

In this study, an image-processing scheme was proposed to improve the visibility of defects with the use of LPT. This algorithm not only extracts the effective grayscale range of the phase image, but also sharpens the edges of defects. Fourier phase analysis is conducted to the image sequence to compute the phase map, so that it can be enhanced with the detail enhancement process. The enhancement processing treats the background and detailed components resulting from separation of the raw phase data independently using an edge preserving filter. Then, the effectiveness of various image processing techniques for treating a sequence of infrared images is assessed including the TSR+1st and 2nd derivatives, PPT, PCA, etc. Moreover, the defect size is measured in the reconstructed thermal image. The process is applied to a polymer composite panel with model defects. Results of the defect detection are presented, and relative advantages and disadvantages of the methods are discussed.

The rest of this study is organized as follows: Section 2 presents the proposed algorithm, which consists of Fourier phase analysis in Section 2.1 and the enhancement of the thermal phase in Section 2.2. Section 3 presents the details of the experiment for investigating the performance. Section 4 gives the experimental results and makes some discussions on the results. Section 5 concludes.

## 2. Methodology

### 2.1. Fourier Phase Analysis

The application of LPT requires the surface of a sample to be heated by a low-power source, typically a pair of halogen lamps for a certain period of time. A sequence of thermal images is recorded with an infrared camera during the cooling period right after thermal excitation. The temperature variation with time at the tested surface can be described by integrating multiple pulse thermal responses since the heat transfer in the long pulse excitation can be treated as the superposition of a series of flash excitation [17]. The surface temperature *T* as the function of time *t* can be expressed as Equation (1) if a defect is located at a distance of *d* from the surface.

(1) $$T\left(t\right)=\int_{0}^{t_{p}}\frac{W}{\rho Cd}\{1+2\sum_{n=1}^{\infty }e^{-\frac{n^{2}\pi^{2}\alpha \left(t+\tau \right)}{d^{2}}}\}d\tau$$

where *t_p_* is the heating time duration of the long pulse, *W* is the heat flux on the specimen surface, *t* is the cooling time, *α* is the thermal emissivity of the sample, *ρ* and *C* are the density and heat capacity of the material, respectively. As the temperature distribution at the testing surface is generally not uniform before thermal radiation due to the environmental conditions, a reference image is expected to calibrate the initial temperature distribution by subtracting from the rest in the sequence. In this study, the reference image is constructed from the temporal average of ten frames captured before heating.

Since the signal-to-noise ratio (SNR) of phase data is higher than the raw temperature data—especially for deep defects—a Fourier phase analysis (FPA) was considered in this study. A one-dimensional Fourier transform was adopted to convert the temperature from the time domain into the frequency domain pixel-by-pixel. The phase image *φ_k_* at frequency *k* was computed as follows:

(2) $$\phi_{k}=\tan^{-1}\frac{\sum_{n=1}^{N}T(n)\cos(2\pi kn/N)}{\sum_{n=1}^{N}T(n)\sin(2\pi kn/N)}$$

where *T(n)* was the temperature at the pixel point (*x, y*) in the *n*_th_ image in the sequence, subscript *k* is the serial number of the discrete frequency and *N* corresponded to the total number of the thermal images acquired.

**Figure 1:**
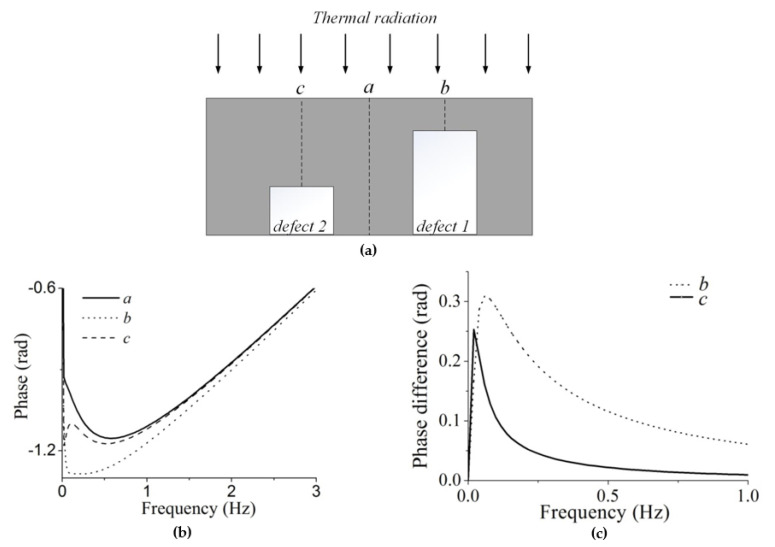
(**a**) Schematics of a panel with defects; (**b**) phase variation at locations with and without defects; (**c**) variation of phase difference corresponding to locations with defects.

As the phase magnitudes in the defective area are much smaller than those in the surrounding area [18], phase difference between the defective and sound regions is evident to determine the locations with defects. A schematic diagram of a panel with defects at different depths is shown in Figure 1a. On the top surface, point *a* indicates a region without defects, while points b and c indicate regions which contain defects with different depths, i.e., defect 1 and defect 2. The phase variations with frequency at points a, b and c in the Fourier domain are illustrated in Figure 1b. The resulting phase differences which are obtained by subtracting the abnormal phase from the normal phase are displayed in Figure 1c. The magnitude of phase differences starts to hike and reaches the maximum at a certain frequency. Interestingly, on each curve there is a peak value in phase difference which can be used to determine the defect information with the best signal to noise ratio. The frequency is considered to be closely related to the depth of defects. In applications, defects can only be detected at low frequencies, because the phase noises increase largely at high frequencies. The peak value turns out to be small and the corresponding frequency is also small if the defect is deep (defect 2). This implies that it is difficult to determine all defects at different depths using a certain phase image.

### 2.2. Enhancement of Thermal Phase

In order to visualize the distribution of phase, a common practice is to convert the phase data to an 8-bit image. For example, the phase map is usually linearly stretched by defining the maximum phase as 255 and the minimum phase as 0. Nevertheless, the phase obtained from the Fourier transform is with high precision. Moreover, such conversion may potentially lead to reduction of image qualities. To solve this problem, an image enhancement strategy is proposed. The total process includes separation of the phase image, edge enhancement and final adjustment of the image. In order to produce high fidelity data, the float phase is converted to a digital image with the grayscale level more than 8 bit. If the phase *P* is calculated by Fourier transform, it can be converted to an *M*-bit monochromatic image according to Equation (3).

(3) $$I_{\left(i,j\right)}=(2^{M}-1)(\frac{P_{\left(i,j\right)}-P_{\min}}{P_{\max}-P_{\min}})$$

where *i* and *j* are the coordinate of a pixel, *P*_min_ and *P*_max_ are the maximum and minimum values in the phase diagram, respectively.

It is worth mentioning that Equation (3) is the standard linear enhancement scheme. To avoid loss of detail information of the phase due to truncation in the conversion, the integer grayscale levels are expected to be large enough. In this study, *M* is set as 16 so that totally 65,536 grayscale levels are involved to represent the phase obtained from FPA.

The clear edge information is always welcome in thermography detection. Since the phase image can be decomposed as the background image and detail layer, the contrast of the latter is critical in display. Thus, in this study the 16-bit phase image *I* is separated into the background *B* and detail images by employing the edge preserving filter [19]. An objective function is established in the subset *w.*

(4) $$min\sum_{i\in w}{\left(I_{i}-B_{i}\right)}^{2}+\frac{\varepsilon }{\left|\nabla I_{i}\right|}{\left|\nabla B_{i}\right|}^{2}$$

where *ɛ* is a regularization parameter and ▽ is the gradient operator. Note that because the gradient of phase image *I*, i.e., |▽*I*| becomes remarkable at the edges of defects, the term $\frac{\varepsilon }{\left|\nabla I_{i}\right|}$ is small. It requires *B* to be equivalent to *I* in order to minimize Equation (4). In this condition, the detail information about the edges is preserved in the background image *B*.

At the area without defect edges, the small gradient of phase image *I* potentially results in a large magnitude of $\frac{\varepsilon }{\left|\nabla I_{i}\right|}$. It requires image *B* to be as smooth as possible, so that the term |▽*B*| is small to minimize Equation (4). The smooth *B* means the background contains low-frequency components of the phase image *I*. The background image *B* is assumed as a linear function of *I*:

(5) $$B_{i}=a_{w}I_{i}+b_{w}\;\;\;i\in w$$

where *a_w_* and *b_w_* are coefficients. By substituting Equation (5) into Equation (4), the objective function about the phase image *I* is rewritten as

(6) $$min{\sum_{i\in w}\left(I_{i}-a_{w}I_{i}-b_{w}\right)}^{2}+\varepsilon \left|\nabla I_{i}\right|a_{w}^{2}$$

The least square method can be adopted to estimate the coefficients in Equation (5):

(7) $$\begin{array}{l} a_{w}=\frac{\sigma_{w}^{2}}{\sigma_{w}^{2}+\frac{\varepsilon }{N}\sum_{i\in w}\left|\nabla I_{i}\right|}\\ b_{w}=\mu_{w}-a_{w}\mu_{w} \end{array}$$

In the subset *w*, *σ_w_*^2^ is the variance of *I*, *N* is the number of pixels and *μ_w_* is the mean value of *I*. In a subset *w*, a pair of *a_w_* and *b_w_* is determined. As a pixel can be involved in multiple subsets, the final coefficients *a* and *b* for such pixel are resulted from averaging all pairs of *a* and *b* involving the pixel. Thus, the background image *B* is accordingly constructed based on Equation (5). By subtracting the background image *B* from the phase image *I*, the portion which describes the edge information of defects is selectively preserved while the rest is filtered. The detail image *D* is constructed according to Equation (8).

(8) $$D_{i}=I_{i}-B_{i},\;\;\;\forall i\in w_{k}$$

By conducting the edge-preserving filter, the Fourier phase image is separated into the background and detail images. Distinct processing approaches are developed for these two images.

As the high-frequency information about the defect edges is mainly involved in the detail image, a process of gaining contrast is expected. It is found that the coefficient *a_w_* in Equation (6) is close to zero at the smooth regions while the maximum at edges. Thus, the detail image *D* can be enhanced by multiplying *a_w_* and a magnification factor *λ*, as expressed in Equation (9).

(9) $$D^{\prime }=\lambda a_{w}\cdot D$$

In practice, the parameter ɛ and subset size play an important role to the signal-to-noise ratio. The empirical rule indicates that a small subset size and a large magnitude of ɛ are helpful in reducing image noises and highlighting edges. This process selectively highlights the edge while suppressing the rest information. In this study, the subset size is chosen as 3 × 3 pixels and *ɛ* is set as 10^4^.

As it contains majorities of the low-frequency information in a large dynamic range in the background image *B*, the histogram projection method is used to compress the wide grayscale levels. A common practice is to define a threshold *T* as 0.1% of the total number of the pixels in the image. By counting the number of pixels at a certain grayscale, an indicator function is deduced as follows

(10) $$H(x)=\left\{ \begin{array}{ll} 0_{} & n_{x}<T\\ 1_{} & n_{x}>T \end{array}\right.$$

where *n_x_* is the number of pixels with the grayscale *x*. The grayscale level x is valid only if *H(x)* equals 1. The cumulative histogram of the background image *B* is expressed as:

(11) $$C(x)=\left\{ \begin{array}{ll} 0_{} & x=0,\\ {\frac{\sum_{y=0}^{x-1}H(y)}{n_{valid}}}_{} & otherwise, \end{array}\right.$$

where *n_valid_* is the total number of the valid grayscales. For display purposes, an 8-bit grayscale image for the background image *B* is recovered by converting grayscales according to

(12) $$B^{\prime }=255*C(x)$$

The updated phase image *O* after the background compression and detail enhancement is obtained by adding *D’* and B*’* according to Equation (13).

(13) $$O=D^{\prime }+B^{\prime }$$

Gamma correction is conducted as the final adjustment to improve the contrast and brightness. The updated phase image *O* is normalized with quantities between 0 and 1 as described by

(14) $$\begin{array}{ll} O^{\prime }(i,j)= & \left\{ \begin{array}{cc} 0_{} & if\;\;\;O(i,j)<\nu_{\min}\\ 1_{} & if\;\;\;O(i,j)>\nu_{\max}\\ {\frac{O(i,j)-\nu_{\min}}{\nu_{\max}-\nu_{\min}}}_{} & other \end{array}\right.\\ & \nu_{\max}=mean\_I+3dev\_I\\ & \nu_{\min}=mean\_I-3dev\_I \end{array}$$

where *i* and *j* are the abscissa and ordinate of the pixel, and *mean_I* and *dev_I* are the mean and standard deviations of the phase image *O*. According to Weber’s law, it is hard for the human vision to distinguish small differences in brightness when the background is too dark or bright, while the same difference in brightness is likely detected in a moderate background. Thus, the gamma value γ is calculated from the background image *B’* according to Equation (15).

(15) $$\gamma (i,j)=\max(\exp(\frac{B^{\prime }(i,j)-128}{128}),1)$$

The final normalized 8-bit phase image *O”* is resulted from the transform described in Equation (16).

(16) $${O^{\prime }}^{\prime }(i,j)=255\cdot O^{\prime }{\left(i,j\right)}^{\gamma (i,j)}$$

The process described above provides an effective way to visualize the defects with clear edges in the phase image. It is applied to enhancement of the series of the phase images resulting from FPA after acquisition of the raw infrared image sequence. As shown in Figure 1, deep defects in the material usually reflect a peak phase difference at a low frequency, while subsurface defects reflect a peak value at a high frequency. For defects at the maximum phase difference, a clear edge with best contrast is obtained with the proposed method. However, with the change of frequency, the edges of defects become blur due to loss of signal-to-noise ratio. As a result, clear defects at a specific depth can only be detected in one phase image. In order to achieve a complete evaluation, a group of images that contains defects at multiple depths is often wanted. In this situation, discrete wavelet transform [20] is a useful tool to form an ultimate image with full information of defects at a good quality.

## 3. Experiments

**Figure 2:**
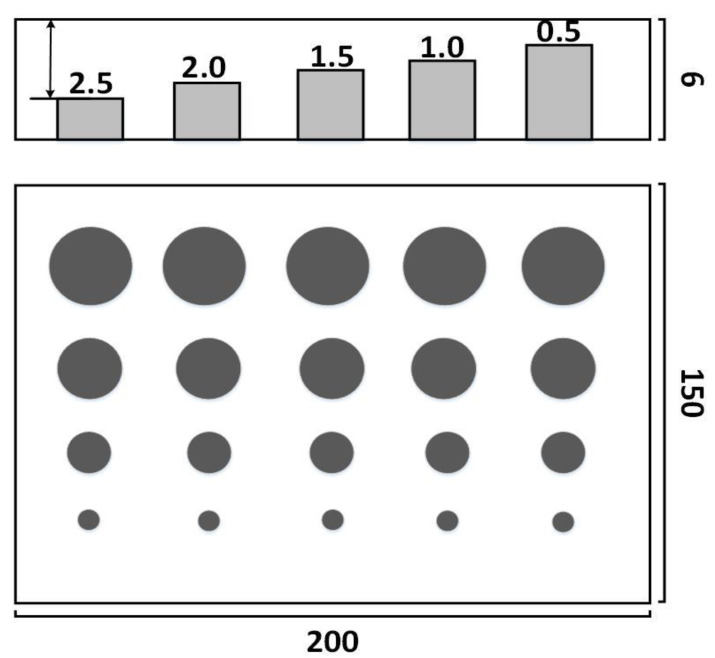
Schematics of defect distribution in the glass-fiber-reinforced plastic (GFRP) panels.

A glass-fiber-reinforced plastic (GFRP) panel with dimensions of 200 mm × 150 mm × 6 mm was prepared. Flat-bottomed holes with diameters from 5 to 20 mm and depths from 0.5 to 2.5 mm were machined. The schematics of defects arrangement in the GFRP panel is shown in Figure 2. The front surface of the specimen was painted with black paint of matt finishing to improve the optical energy absorption and emissivity at the surface.

In this work, two commercially available halogen lamps were employed to achieve long pulse thermal excitation since this was a cheap and easy to provide uniform and repeatable heating for the quantitative examination. The quartz halogen lamps in this illustration were far less expensive than the special 6-kJ flash lamps used in PT and it does not require accurate synchronization of excitation between flashing and IR imaging [5]. In the experiment, two halogen lamps with an individual power of 1000 W were placed at a distance of about 1000 mm in front of the panel. The point light source was expanded with a semispherical back aluminum cover and scattered onto the panel surface. A mid-wave infrared camera (FLIR A6700SC, FLIR Systems, USA), which is sensitive to 3–5 µm wavelength, was used to capture sequential images right after the heating process. The heating duration was set to 10 s. In order to study the effect of experimental parameters such as sampling rate on image quality, multiple sequential images were acquired right after heating.

## 4. Results and Discussion

### 4.1. Comparison of Thermal Signal Reconstruction Methods

**Figure 3:**
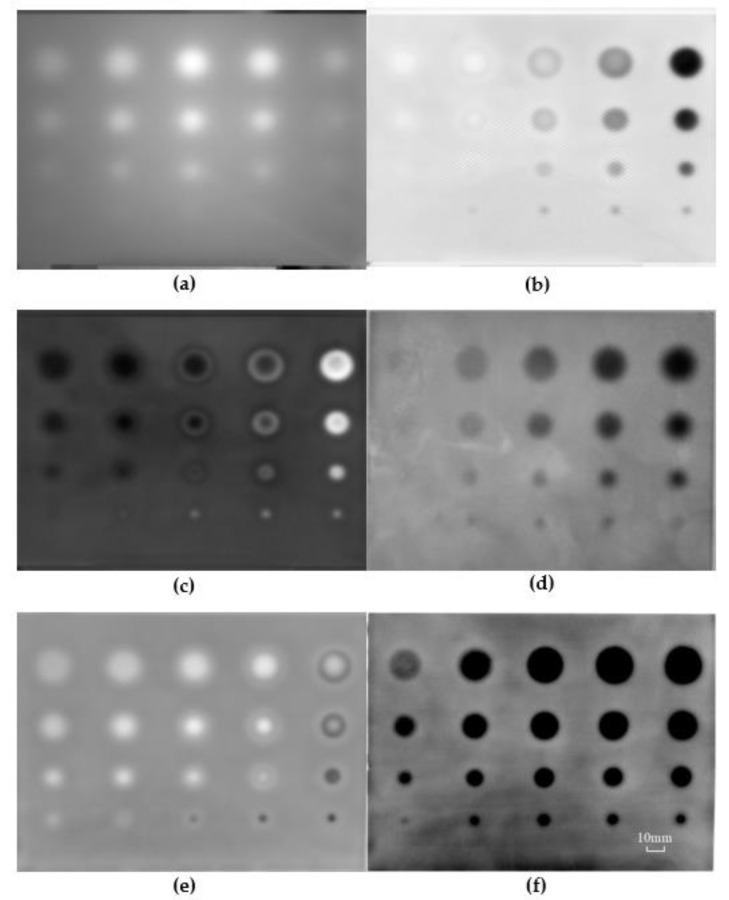
Detected images with varied methods in the GFRP panel. (**a**) Raw image; (**b**) thermographic signal reconstruction (TSR)+1D, (**c**) TSR+2D; (**d**) pulse phase thermography (PPT); (**e**) principal component analysis (PCA); (**f**) proposed method.

The inspected thermal images for the GFRP panel are shown in Figure 3. As an example, a representative of the raw thermographs corresponding to the 270^th^ image with the sampling frequency at 10 Hz is shown in Figure 3a. Although the image possesses good quality in the raw sequences, the defects with the smallest diameter can hardly be perceived. In addition, the edges of the defects with the maximum and minimum depths are blurred, which potentially contributes to errors in measuring defect geometries.

In order to evaluate the effectiveness of the proposed image enhancement algorithm, some existing thermal signal processing methods including TSR derivative, PPT and PCA were employed in processing the image sequence acquired in the span of 51.2 s with a frequency of 10 Hz. The result processed with the TSR+1st derivative (TSR+1D) of the infrared image is shown in Figure 3b. Three columns of the defects on the right side were visible. However, the rest of the defects with depths of 2.0 and 2.5 mm were not identified clearly. As these defects are deep, the effect of diffusion in heat conduction results in very subtle differences in temperature variation between the defects and surrounding sound regions. From this point of view, the TSR+1st derivative image could only visualize defects with large size close to the surface. The result processed with the TSR+2nd derivative (TSR+2D) of the infrared images is shown in Figure 3c. Compared with the image resulting from TSR+1st derivative, the edges of defects are clearer. Except the two defects on the left column, the remaining defects are able to be measured quantitatively. Nevertheless, some defects exhibit false edges due to the disadvantage of the 2nd derivative process. Furthermore, the TSR+1st and the TSR+2nd derivative methods are time-consuming on the thermal data fitting and logarithmic computing. The order of the polynomial fitting is usually difficult to be determined, which potentially influences the quality of the inspection images. The sequential images were also processed with PPT. The phase image at the frequency of 0.039 Hz is shown in Figure 3d. Four defects at depth of 2.5 mm in the left column are not visible. The result processed with PCA is shown in Figure 3e. Although all defects in the panel are visible, it consumes considerable computing time. Some fake edges at the right column are also detected, which may potentially lead to errors in determining the size of defects. The image processed using the proposed algorithm is shown in Figure 3f. The experimental results show that the proposed method results in images with crisper edges and is helpful in identifying small defects. It is evident that this algorithm can the substantially suppress noises and improve visibility of defects with sharp edges.

**Figure 4:**
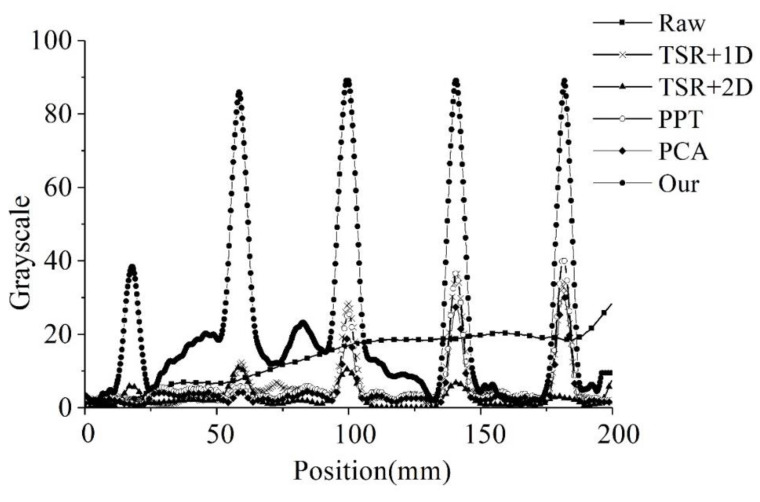
Grayscale profile in a horizontal line for the 5 mm defects.

The grayscale profiles of a horizontal line in the detected images resulted from the selected processing methods are shown in Figure 4. With the use of the proposed method, a fairly good contrast was achieved even for the defect with depth of 2.5 mm and diameter of 5 mm. The size of defects was determined by measuring the grayscale profile directly from the thermal enhanced images in Figure 3f. This is a simple and efficient method for defect sizing. The diameters of the defects were estimated as listed in Table 1. Thanks to the clarity of the enhanced image with sharp edges, the error in measuring defect size for the GFRP was about 2.5%. This experiment provides convincing evidence that the proposed algorithm is effective and accurate in determining the defect edges.

A quantitative evaluation of SNR about the processed images is necessary to assess the merits of different algorithms [21].

(17) $$SNR=\frac{\left|S_{defect}-S_{sound}\right|}{\sigma_{sound}}$$

In this equation, S*_defect_* and S*_sound_* are the average of grayscales over the entire area of the defect and sound regions, respectively; the term σ*_sound_* is the standard deviation of grayscales in the sound area. The SNRs calculated from the images shown in Figure 3 are listed in Table 2. It is obvious that the proposed method is able to achieve a high-SNR image, which is very helpful in image based nondestructive evaluation. Furthermore, the computation time of the selected methods was also listed in Table 2. The calculation time of the proposed method is slightly longer than that of the PPT method because of the additional image enhancement operation. However, the proposed algorithm significantly improved the SNR from 3.27 to 13.33. By contrast, the calculation time of both TSR methods was more than one minute due to the adoption of the time-consuming, pixel-by-pixel one-dimensional polynomial fitting. The PCA method was the worst case due to the use of the singular value decomposition (SVD) in converting the 3-D thermal data to a 2-D image. The results show that the proposed method is capable of improving the reconstruction quality of the thermal signal at a low computational load clearly.

### 4.2. The determination of Parameters in the Proposed Method

In the proposed method, the Fourier phase analysis is employed to convert the temperature variation to the phase variation at the object surface. In order to maintain the trivial difference at the defect edge, an *M*-bit monochromatic image is constructed according to Equation (3). Intuitively, the larger *M* is selected, the bigger differences in grayscales can be found at the edge. However, a large *M* may raise efficiency concerns in image processing. In this study, this parameter is considered by setting as 10, 16 and 20. The image quality is evaluated with the contrast-per-pixel (CPP) index [22], which is defined as follows.

(18) $$\mathrm{CPP}=\frac{\sum_{i=1}^{r}\sum_{j=1}^{c}\left(\sum_{m,n=1}^{1}\left|Im(i,j)-Im(i+m,j+n)\right|\right)}{r\times c}$$

In the equation, *Im(i,j)* is the grayscale at pixel (*i,j*); the parameters *r* and *c* are the height and width of the image. The quantity of the CPP index reflects the contrast of the image. Assume 512 frames of phase images with spatial resolution of 640 × 512 pixels are generated from phase calculation. As indicated in Table 3, the CPP index is considerably improved if M changes from 10 to 16. At the same time, the memory size and computing time increase accordingly in this situation. It is worth noting that the CPP index does not increase much if M changes from 16 to 20 while the computing cost increases. Thus, M = 16 can be considered as an optimal for image conversion.

**Figure 5:**
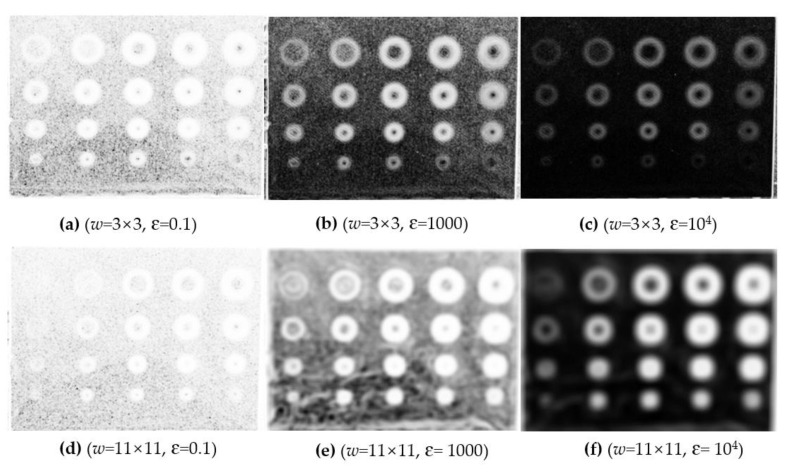
Coefficient images *a_w_* with different subset windows and ɛ.

In the proposed method, the phase image is divided into the detail and background images. The detail image which contains information of defect edges is enhanced according to Equation (9). Since the quality of *a_w_* is mainly determined by the regularization parameter *ɛ* and subset size *w* according to Equation (7), the parameter ɛ and subset size *w* are key factors which influence the signal-to-noise ratio of the processed image. Considering the typical spatial resolution of an infrared image as 640 by 512 pixels, the subset size *w* was defined as 3 × 3 or 11 × 11, and the regularization parameter *ɛ* was enumerated as 0.1, 1000, and 10,000. Illustrations of *a_w_* are provided with multiple combinations of parameter ɛ and subset size *w*, as shown in Figure 5. From the left to right, it is found that the increase of quantity of ɛ results in a high contrast at the defect edges and is helpful in improving the SNR in the inspection image. Comparing the first and the second rows, a small subset size *w* results in clear defect edges, while a large subset size *w* blurs the defect edges. Therefore, it is reasonable to choose a small subset size *w* to preserve defect details and a large quantity of ɛ to improve contrast between the sound and defect areas. From this point of view, it is a wise strategy by setting ɛ = 10^4^ and *w* = 3 × 3 in the detail image enhancement. In addition, the CPP index is the maximum if this combination is selected as shown in Table 4.

The proposed method is efficient in identifying defects at multiple depths with clear edge. It separates the digitized phase image into the background image and the detail image. The detail image contains information about the edges of defects, and it can be enhanced according Equation (9) by using an appropriate subset size and a large ɛ magnitude. These processes achieve a high contrast between defects and sound areas which results in a clear border in between.

### 4.3. The Effect of Sampling Rate

The sampling rate is a critical factor that influences the detection results. A large number of images can be collected at a high sampling rate within a given time. Although it helps to record a continuous change in thermal variations on the specimen surface, which is substantially beneficial to the Fourier phase analysis, it requires a large memory for data storage and sacrifices computing efficiency. On the contrary, sequential images with low sampling rate may miss information of variations of temperature, and potentially result in images with fuzzy defect edges using the proposed method. Therefore, a moderate sampling rate should be determined to balance the image quality and the computing efficiency.

**Figure 6:**
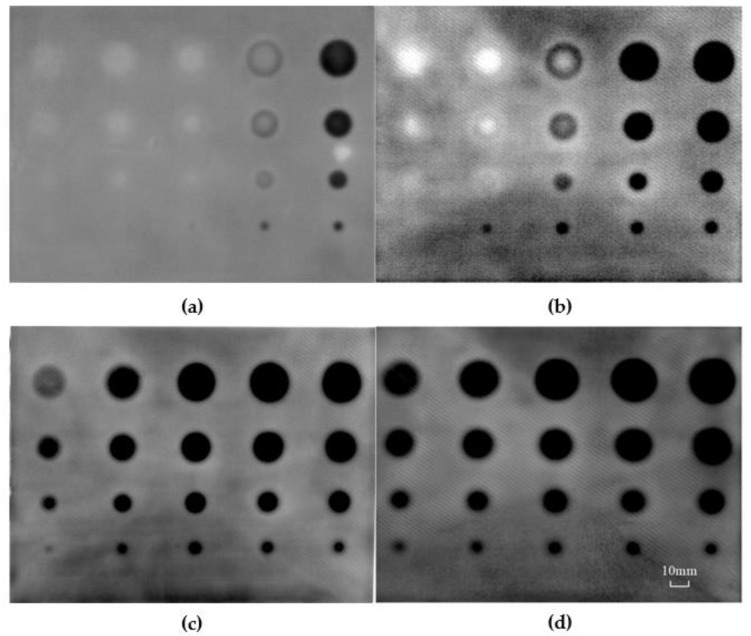
Detected images with varied image sampling rate in the GFRP panel. (**a**) 1 Hz; (**b**) 5 Hz; (**c**) 10 Hz; (**d**) 30 Hz.

As an illustration, the sequential infrared images of the GFRP panel were recorded at multiple sampling rate including 1, 5, 10 and 30 Hz, after the panel was heated for 10 s. The total acquisition time was 51.2 s. It was found that all 20 defects became clear in the detected images with a sampling rate of 10 Hz or 30 Hz in Figure 6, while some defects were blurred at edges with low the sampling rates.

**Figure 7:**
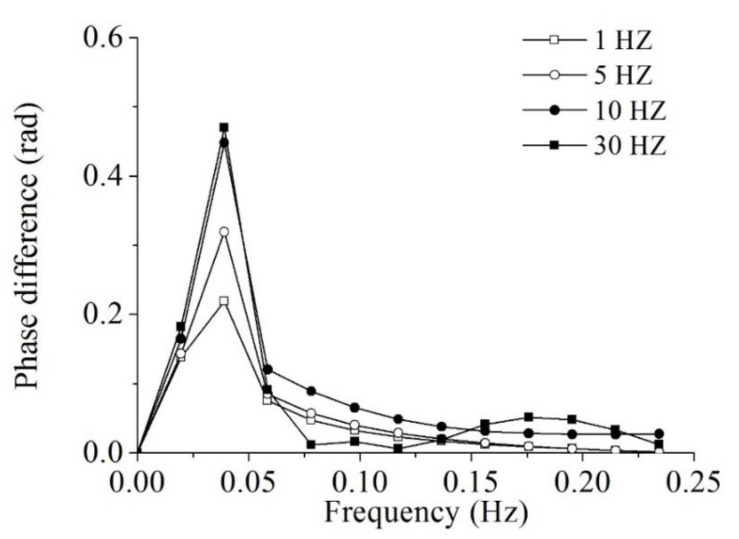
Phase difference variations for different sampling rates.

The corresponding Fourier phase difference vs. frequency curves are shown in Figure 7. It was evident that the maximum phase difference (peak value) corresponding to each sampling rate increased with the sampling rate. When the sampling rate increased from 1 Hz to 5 Hz and from 5 Hz to 10 Hz, the defects in the resulting images in Figure 6 became clearer with the maximum phase difference increased from 0.21 to 0.32 and from 0.32 to 0.45 (Figure 7). Thus, to raise the sampling rate is helpful to improve the image quality. However, the peak value of the phase difference only varied from 0.45 to 0.47 when the sampling rate changed from 10 Hz to 30 Hz. It occupied more data space at 30 Hz, while the improvement of image is not appreciable. Considering the balance of detection quality and computing efficiency, the sampling rate at 10 Hz was considered as the optimal solution for the proposed method.

## 5. Conclusions

In this study, a novel thermal signal processing approach that consists of Fourier phase analysis and image enhancement using edge preserving filter and gamma correction was developed for applications with long pulse thermography. The inspection system uses common halogen lamps as the excitation source to heat the object surface for a few seconds and an infrared camera acquires images while the object is cooling down. A unique aspect of the proposed method is to split the digitized phase image into a background image (B), which contains low frequency component and a detailed image (D)*,* which preserves small intensity variations and enhanced by the linear constant coefficients *a_w_*. This approach can appreciably enhance the edge of defects and improve visibility of defects. The proposed method was applied to inspection of the glass-fiber-reinforced polymer composite panel with predefined defects, where thermal images were recorded by an infrared camera. The experimental results demonstrate that the simple thermography system along with the developed algorithm is effective for nondestructive evaluation of composite structures.

## Figures and Tables

**Table 1:** Comparison of the Flaw Diameters with Measured and Designed.

Flaw	Measured (mm)	Designed (mm)	Error (%)
1	5.2	5	5.2
2	9.9	10	1.0
3	15.2	15	1.3
4	20.5	20	2.5
			Average = 2.5%

**Table 2:** Comparison of the Signal-to-Noise Ratio (SNR) and the Computational Time for the Proposed and Existing Methods.

Method	SNR (dB)	Computational Time (s)
TSR+1D	2.69	70.357
TSR+2D	7.78	67.073
PPT	3.27	18.510
PCA	9.13	96.881
Our	13.33	24.548

**Table 3:** Comparison of the Contrast-Per-Pixel (CPP) Index and Computational Time with Different Grayscale Levels.

M CPP Index		Memory Consumption (MB)	Computational Time (s)
10bit	18.97	200	20.346
16bit	66.01	320	24.548
20bit	70.23	400	33.447

**Table 4:** Comparison of the CPP Indices with Different Parameter Combinations.

*ɛ*	*w*	CPP Index
0.1	3×3	40.75
1000	3×3	63.70
10,000	3×3	66.01
0.1	11×11	11.86
1000	11×11	12.09
10,000	11×11	11.25
